# Acidity of persulfides and its modulation by the protein environments in sulfide quinone oxidoreductase and thiosulfate sulfurtransferase

**DOI:** 10.1016/j.jbc.2024.107149

**Published:** 2024-03-11

**Authors:** Dayana Benchoam, Ernesto Cuevasanta, Joseph V. Roman, Ruma Banerjee, Beatriz Alvarez

**Affiliations:** 1 Laboratorio de Enzimología, Instituto de Química Biológica, Facultad de Ciencias, Universidad de la República, Montevideo, Uruguay; 2 Centro de Investigaciones Biomédicas (CEINBIO), Universidad de la República, Montevideo, Uruguay; 3 Graduate Program in Chemistry, Facultad de Química, Universidad de la República, Montevideo, Uruguay; 4 Unidad de Bioquímica Analítica, Centro de Investigaciones Nucleares, Facultad de Ciencias, Universidad de la República, Montevideo, Uruguay; 5 Laboratorio de Fisicoquímica Biológica, Instituto de Química Biológica, Facultad de Ciencias, Universidad de la República, Montevideo, Uruguay; 6 Department of Biological Chemistry, University of Michigan Medical School, Ann Arbor, Michigan, USA

**Keywords:** persulfide, thiol, alpha effect, sulfide quinone oxidoreductase, thiosulfate sulfurtransferase, p*K*_a_, rhodanese, hydrogen sulfide

## Abstract

Persulfides (RSSH/RSS^−^) participate in sulfur metabolism and are proposed to transduce hydrogen sulfide (H_2_S) signaling. Their biochemical properties are poorly understood. Herein, we studied the acidity and nucleophilicity of several low molecular weight persulfides using the alkylating agent, monobromobimane. The different persulfides presented similar p*K*_a_ values (4.6–6.3) and pH-independent rate constants (3.2–9.0 × 10^3^ M^−1^ s^−1^), indicating that the substituents in persulfides affect properties to a lesser extent than in thiols because of the larger distance to the outer sulfur. The persulfides had higher reactivity with monobromobimane than analogous thiols and putative thiols with the same p*K*_a_, providing evidence for the alpha effect (enhanced nucleophilicity by the presence of a contiguous atom with high electron density). Additionally, we investigated two enzymes from the human mitochondrial H_2_S oxidation pathway that form catalytic persulfide intermediates, sulfide quinone oxidoreductase and thiosulfate sulfurtransferase (TST, rhodanese). The pH dependence of the activities of both enzymes was measured using sulfite and/or cyanide as sulfur acceptors. The TST half-reactions were also studied by stopped-flow fluorescence spectroscopy. Both persulfidated enzymes relied on protonated groups for reaction with the acceptors. Persulfidated sulfide quinone oxidoreductase appeared to have a p*K*_a_ of 7.8 ± 0.2. Persulfidated TST presented a p*K*_a_ of 9.38 ± 0.04, probably due to a critical active site residue rather than the persulfide itself. The TST thiol reacted in the anionic state with thiosulfate, with an apparent p*K*__a__ of 6.5 ± 0.1. Overall, our study contributes to a fundamental understanding of persulfide properties and their modulation by protein environments.

Persulfides are compounds with the general formula RSSH/RSS^−^. Unlike thiols (RSH/RS^−^) and hydrogen sulfide (H_2_S/HS^−^), persulfides possess a sulfane sulfur atom, that is, a sulfur bonded to either two sulfurs or to a sulfur and an ionizable hydrogen (1). The term *persulfide* is used in this text for the mixture of hydropersulfide (RSSH) and persulfide anion (RSS^−^) in aqueous solution.

Prominent roles have been assigned to persulfides in biological systems; they participate in sulfur trafficking, biosynthesis and catabolism, and are considered potential transducers of the beneficial physiological effects of H_2_S in mammals (2, 3, 4). They are endogenously synthesized through H_2_S-dependent and H_2_S-independent pathways. The reaction of H_2_S with an oxidized thiol derivative, such as disulfide, sulfenic acid, or trisulfide, gives a persulfide in addition to a thiol, water, or another persulfide, respectively (5). Additionally, thiols can react with oxidized derivatives of H_2_S, such as thiosulfate (SSO_3_^2−^), persulfides, and polysulfides (HS_n_SH, RS_n_SSH, RS_n_SSR, n ≥ 1) to form persulfides (6). The transfer of sulfur from persulfides to thiols to form new persulfides at the attacking thiol is called transpersulfidation. Other routes for persulfide formation involve free radical–mediated processes (7). Regarding H_2_S-independent pathways, there are several enzymes capable of producing persulfides with sulfur donated by thiols or disulfides (8, 9, 10). Persulfides can occur in low molecular weight (LMW) compounds as well as in cysteine residues of proteins. In this sense, micromolar levels of glutathione persulfide (GSSH), cysteine persulfide (CysSSH), and protein persulfides have been reported (10, 11, 12).

The extra sulfur in persulfides in comparison with thiols confers unique properties. Protonated persulfides (RSSH) ionize in aqueous solution to give the corresponding anionic species (RSS^−^). LMW persulfides have been found to be more acidic than the analogous thiols due to their weaker S-H bond (13, 14, 15). For example, the apparent p*K*_a_ of GSSH is 5.45 (13) while that of glutathione (GSH) is 8.94 (16). Thus, at physiological pH, GSSH is almost completely ionized to GSS^−^ but GSH is mostly protonated. Moreover, persulfides possess enhanced nucleophilicity compared to thiols at physiological pH, which results from the combination of two factors. The first factor is the availability of the anionic species (which is a better nucleophile than the protonated one), and the second factor is the high nucleophilic reactivity of the anionic species due to the alpha effect (13), which is caused by the presence of high electron density in the atom adjacent to the nucleophilic atom (17, 18). Furthermore, unlike thiols, persulfides are also electrophilic. Both sulfur atoms are susceptible, and depending on the site of the nucleophilic attack, H_2_S or thiol is eliminated. Given the dual reactivity, persulfides decay in aqueous solution. Hence, *in vitro* preparations of LMW persulfide also contain other species such as thiols, H_2_S, disulfides, and polysulfides (13, 19). Considering that electrophilicity is mainly ascribed to the protonated species, whose abundance is predicted to be low at physiological pH based on the low p*K*_a_s reported for LMW persulfides, persulfides are expected to play roles as nucleophiles in biological systems, as in the reaction between GSSH and the enzyme persulfide dioxygenase (or ETHE1). However, some protein persulfides play prominent roles as electrophiles, for example, in the catalytic cycles of the mitochondrial H_2_S oxidation enzymes, sulfide quinone oxidoreductase (SQOR, EC 1.8.5.8), and thiosulfate sulfurtransferase (TST, also called rhodanese, EC 2.8.1.1).

SQOR catalyzes the first step of the H_2_S oxidation pathway in mitochondria. This step consists of the transfer of sulfur from H_2_S to a LMW thiophilic acceptor with the concomitant formation of reduced coenzyme Q_10_ (CoQ_10_), which then enters the electron transport chain (20). Human SQOR is a flavoenzyme with a cysteine trisulfide (Cys_379_-S-S-S-Cys_201_) in the active site (21, 22). Three substrates are involved in its activity; H_2_S as sulfur donor, a sulfur acceptor, and CoQ_10_. The proposed mechanism (21) begins with the attack of HS^−^ on the trisulfide to form two persulfides, one at Cys_379_ and another at Cys_201_, which forms a transient charge transfer (CT) complex with the FAD cofactor (Fig. 1*A*, reaction *a*). The persulfide in Cys_379_ is attacked by a thiophilic acceptor that extracts the sulfane sulfur and releases a thiolate in Cys_379_, while the CT complex at Cys_201_ is presumed to evolve to a C4a covalent adduct (Fig. 1*A*, reaction *b*). The thiolate in Cys_379_ attacks the adduct, regenerating the trisulfide and producing FADH_2_ (Fig. 1*A*, reaction *c*). Then, CoQ_10_ is reduced by FADH_2_ to complete the catalytic cycle (Fig. 1*A*, reaction *d*). Regarding the thiophilic acceptor, GSH has been proposed to be the physiologically preferred substrate, which would lead to GSSH formation (23, 24, 25). However, human SQOR exhibits remarkable substrate promiscuity (20). Additional *in vitro* sulfur acceptors include sulfite (SO_3_^2−^), cyanide (CN^−^), a second H_2_S, methanethiol, and coenzyme A, which produce thiosulfate, thiocyanate (SCN^−^), H_2_S_2_, methanethiol persulfide, and coenzyme A persulfide, respectively (20, 21, 23, 24, 25, 26, 27). GSSH can be further converted to sulfite by persulfide dioxygenase at the expense of O_2_ (23, 28) or to thiosulfate by TST at the expense of sulfite (24).

**Figure 1:**
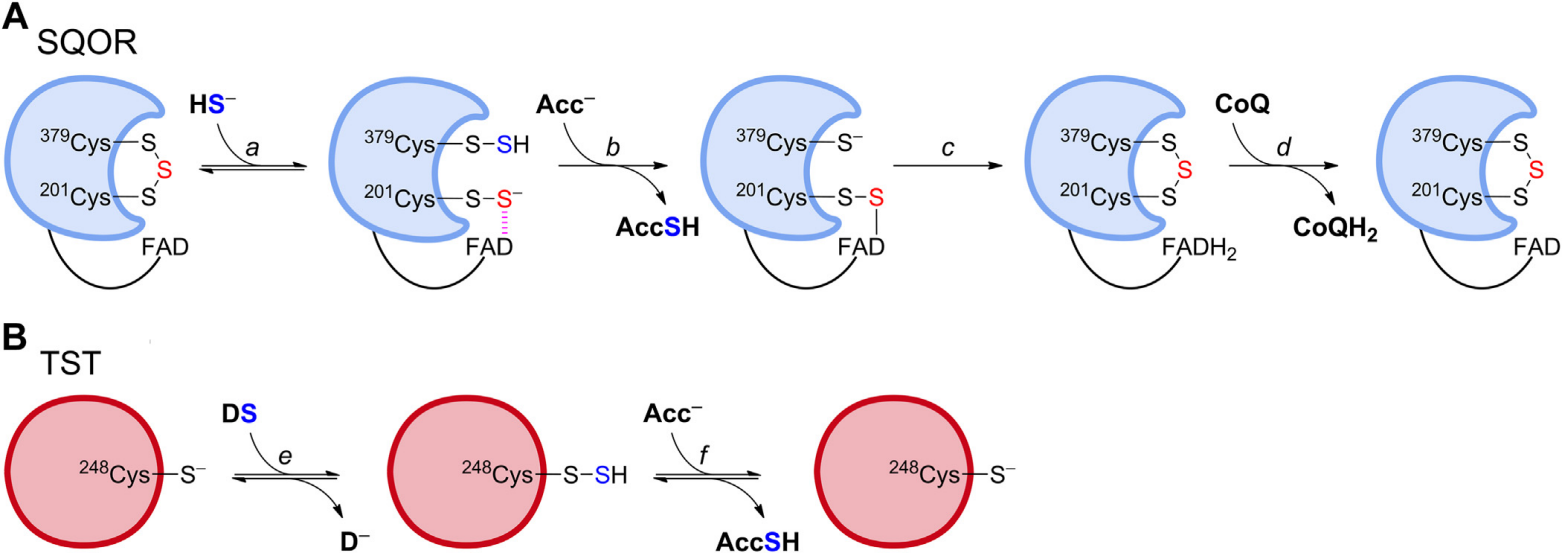
**Proposed catalytic mechanisms for human SQOR and TST.***A*, the active site cysteine trisulfide in SQOR is attacked by H_2_S to form two persulfides, at Cys_379_ and at Cys_201_; the latter engages in a transient charge transfer (CT) complex with the FAD cofactor (reaction *a*). Then, a thiophilic acceptor (Acc^−^) attacks Cys_37__9_SSH and the sulfane sulfur is transferred, generating AccSH and Cys_379_SH while the CT complex is proposed to evolve to a transient C4a adduct (reaction *b*). Then, Cys_379_SH attacks the adduct, regenerating the trisulfide and producing FADH_2_ (reaction *c*). Finally, FADH_2_ reduces CoQ to CoQH_2_, restoring SQOR to its resting state (reaction *d*) (21). *B*, the active site Cys_248_SH in TST attacks a sulfane sulfur donor (DS), resulting in the formation of Cys_248_SSH and release of the first product (D^−^) (reaction *e*). Next, a thiophilic acceptor (Acc^−^) attacks Cys_248_SSH, producing AccSH and restoring the resting enzyme (reaction *f*). SQOR, sulfide quinone oxidoreductase; TST, thiosulfate sulfurtransferase.

TST catalyzes the transfer of a sulfane sulfur from a donor to an acceptor using a ping-pong mechanism. The minimal reaction mechanism comprises a first step of nucleophilic attack by an active site cysteine (Cys_248_) on a sulfane sulfur donor, resulting in the release of the first product and formation of a cysteine persulfide on the enzyme (Fig. 1*B*, reaction *e*). In the second step, the persulfide is attacked by a thiophilic acceptor, releasing the second product and restoring the enzyme to its resting state (Fig. 1*B*, reaction *f*). Possible sulfur donors include thiosulfate and GSSH (producing sulfite and GSH, respectively), while possible sulfur acceptors include cyanide, sulfite, and GSH (generating thiocyanate, thiosulfate, and GSSH, respectively) (23, 24, 29). The best characterized reaction is with thiosulfate and cyanide as substrates. The enzyme mechanism can include additional steps, such as the formation of a noncovalent Michaelis complex with thiosulfate before sulfur transfer (30, 31).

In a previous work, we studied the reactions of GSSH with different electrophiles, leading to the determination of the p*K*_a_ of GSSH and to quantitative evidence for the enhanced nucleophilicity of GSSH compared to that of a putative thiol with the same p*K*_a_, that is, the alpha effect (13). In this work, we extended the investigation to determine the p*K*_a_ values of several LMW persulfides, including the cysteine derivative, CysSSH. Considering that the p*K*_a_ of cysteine as a free amino acid in aqueous solution is different from that of protein cysteine residues, differences are also expected in the p*K*_a_ of free CysSSH compared to persulfides formed in proteins. Thus, we aimed to determine the p*K*_a_ of the persulfides formed at Cys_379_ on human SQOR and at Cys_248_ on human TST. The activities of both enzymes were measured at varying pH using sulfite or cyanide as sulfur acceptors. In addition, the half-reactions between TST persulfide and both sulfur acceptors were studied, representing the first pre-steady state kinetic characterization of TST to our knowledge. Our study elucidates the acidity of various LMW persulfides and reaffirms the alpha effect. It also provides a comparison between free and protein-bound persulfides, revealing that the protein environment can modulate persulfide properties.

## Results

### LMW persulfides

The p*K*_a_ values of several LMW persulfides were studied through the pH-dependency of the reaction rates with the alkylating agent monobromobimane (mBrB), as described previously for GSSH (13). We took advantage that mBrB produces fluorescent products, is uncharged, and does not accept or release protons within the pH range studied. Additionally, mBrB can be used in pseudo-first order excess, abrogating the need to know the exact persulfide concentration.

**Figure 2:**
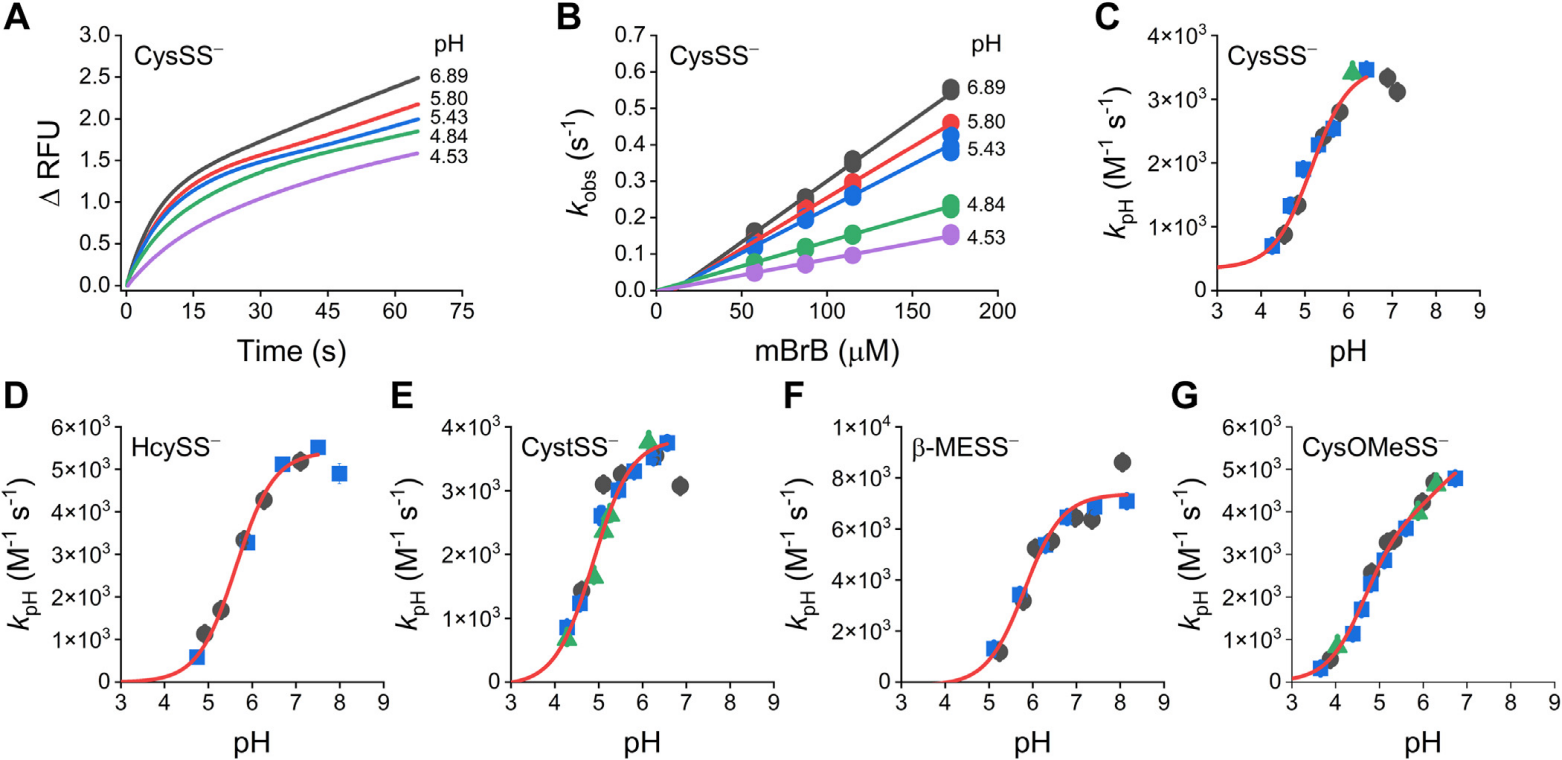
**p*K***_**a**_**of LMW persulfides and their reactivity with mBrB.***A*, representative stopped-flow fluorescence kinetic traces (λ_ex_ = 396 nm, emission cut-off 435 nm) of the reaction of CysSSH-containing mixtures (0.5–3 μM) with mBrB (57.5 μM) in acetic/MES/Tris buffer (pH 3.65–8.15, 25 °C). Exponential plus straight line functions were fitted to the time courses over 10 half-lives. In some cases, where double exponential plus straight line functions were fitted, the exponential phase with the lower observed rate constant (*k*_obs_) and larger amplitude was attributed to the reaction of the persulfide with mBrB. *B*, linear dependence of *k*_obs_ of CysSS^−^ with mBrB concentration. *Circles* are quintuplates of *k*_obs_ obtained for every pH and mBrB concentration. The slope at each pH represents the apparent second-order rate constants, *k*_pH_. At the more alkaline pH values, a small negative *y*-intercept was observed, as seen previously with GSSH (13). *C*, for CysSS^−^, a single-p*K*_a_ function was fitted to the plot of *k*_pH_*versus* pH with data obtained in three independent experiments (*black circles*, *blue squares*, and *green triangles*). A p*K*_a_ of 5.2 ± 0.1 and a pH-independent second-order rate constant, *k*_ind_, of (3.2 ± 0.1) × 10^3^ M^−1^ s^−1^ (parameters ± standard errors of the fit) were determined. As seen previously with GSSH (13), a small decrease of unknown origin in the *k*_pH_ at the more alkaline pH values was observed. *D*–*G*, the p*K*_a_ and *k*_ind_ of the reactions with mBrB for other LMW persulfides were determined analogously. Plots of *k*_pH_*versus* pH for HcySS^−^ (*D*), CystSS^−^ (*E*), β-MESS^−^ (*F*), and CysOMeSS^−^ (*G*). In the case of CysOMeSS^−^, a two-p*K*_a_ function was fitted resulting in two sets of p*K*_a_ and *k*_ind_ values. The obtained values are summarized in Table 1. CysOMeSS^−^, cysteine methyl ester persulfide anion; CysSSH, cysteine persulfide; CystSS^−^, cysteamine persulfide anion; HcySS^−^, homocysteine persulfide anion; LMW, low molecular weight; mBrB, monobromobimane; β-MESS^−^, β-mercaptoethanol persulfide anion.

Mixtures containing CysSSH, homocysteine persulfide (HcySSH), cysteamine persulfide (CystSSH), β-mercaptoethanol persulfide (β-MESSH), or cysteine methyl ester persulfide (CysOMeSSH), produced from H_2_S and the corresponding symmetrical LMW disulfide (13), were exposed to excess mBrB at different pH values at 25 °C. The fluorescent time courses were biphasic; the rapid exponential phases were attributed to the reactions with the persulfides, while the linear phases were attributed to those with the corresponding thiols present in the mixtures and, secondarily, with H_2_S (13). Exponential plus straight line functions were fitted, and observed rate constants (*k*_obs_) were obtained for each pH and mBrB concentration. The *k*_obs_ increased linearly with the concentration of mBrB, indicating that the reactions were first-order in mBrB, yielding apparent second-order rate constants at each pH (*k*_pH_). All persulfides exhibited the same behavior. Representative plots of the reaction with CysSSH are shown (Fig. 2, *A* and *B*). For each persulfide, the *k*_pH_ increased sigmoidally with pH, confirming that the anionic forms react with mBrB (Fig. 2, *C*–*G*). The p*K*_a_ values of the persulfides and the pH-independent rate constants (*k*_ind_) for the reactions with mBrB were determined from these graphs; the *k*_ind_ value corresponds to the rate constant with completely ionized persulfide. Single-p*K*_a_ functions were fitted to the sigmoidal plots, with the exception of the CysOMeSSH data, where a two-p*K*_a_ function was fitted, obtaining two values of p*K*_a_ and *k*_ind_ (Fig. 2*G*).

Our data are summarized in Table 1, together with values reported for other persulfides and for the analogous thiols. The different persulfides had similar p*K*_a_ values, 4.6 to 6.3, and similar *k*_ind_ for the reaction with mBrB, 3.2 to 9.0 × 10^3^ M^−1^ s^−1^.

**Table 1:** p*K*_a_ values of LMW persulfides and the corresponding thiols and *k*_ind_ of the reactions with mBrB, at *I* = 0.15 and 25 °C

Persulfide	Structure	p*K*_a_			*k*_ind_ with mBrB	
		Persulfide	Analog thiol	Δp*K*_a_	Persulfide (× 10^3^ M^−1^ s^−1^)	Analog thiol (M^−1^ s^−1^)
Cysteine methyl ester (CysOMeSS^−^)		4.6 ± 0.1 ^*a*^	7.44 ^*b*^	2.8	3.8 ± 0.4 ^*a*^	N.D.
		6.3 ± 0.6 ^*a*^	8.88 ^*b*^	2.6	5.4 ± 0.8 ^*a*^	N.D.
Cysteamine (CystSS^−^)		4.87 ± 0.09 ^*a*^	8.21 ^*c*^	3.34	3.9 ± 0.2 ^*a*^	N.D.
Cysteine (CysSS^−^)		5.2 ± 0.1 ^*a*^	8.29 ^*d*^	3.1	3.2 ± 0.1 ^*a*^	105 ^*d*^
Glutathione (GSS^−^)		5.45 ± 0.03 ^*e*^	8.94 ^*d*^	3.49	9.0 ± 0.2 ^*e*^	208 ^*d*^
Homocysteine (HcySS^−^)		5.63 ± 0.06 ^*a*^	9.1 ^*d*^	3.5	5.4 ± 0.1 ^*a*^	N.D.
β-Mercaptoethanol (β-MESS^−^)		5.8 ± 0.1 ^*a*^	9.6 ^*d*^	3.8	7.5 ± 0.4 ^*a*^	519 ^*d*^
2-(3-Aminopropyl-amino)ethane		6.2 ± 0.1 ^*f*^	7.6 ^*f*^	1.4	N.D.	N.D.
Cumene		7.0 ^*g*^	>10 ^*h*^	>3	N.D.	N.D.

**Reported data from *^a^* This work, *^b^* (****43****), *^c^* (****63****), *^d^* (****16****), *^e^* (****13****), *^f^* (****15****), *^g^* (****14****), *^h^* predicted value based on similar thiols (****64****)**.

Abbreviation: N.D., not determined.

### Sulfide quinone oxidoreductase

The acidity of the persulfidated SQOR was evaluated by the pH-dependence of the steady-state rates. For enzymes that catalyze reactions with one substrate, the kinetic parameter *k*_cat_/*K*_m_ (specificity constant) is an apparent second-order rate constant that reports on the properties of the free enzyme and the free substrate (32). *k*_cat_/*K*_m_ can be calculated from the kinetic parameters *k*_cat_ and *K*_m_ obtained from typical Michaelis–Menten analysis (Equation 1). Alternatively, *k*_cat_/*K*_m_ can be determined by measuring the steady-state reaction rate at low substrate concentrations and dividing the rate by the enzyme and the initial substrate concentration, [E]_T_ and [S]_T_, respectively (Equation 2) (33).

(1) $$V_{0}=\frac{V_{\max}{\;\left[S\right]}_{T}}{K_{m}+{\;\left[S\right]}_{T}}=\frac{k_{\text{cat}}\;{\;\left[S\right]}_{T}\;{\;\left[E\right]}_{T}}{K_{m}+{\;\left[S\right]}_{T}}$$

(2) $${{\;\text{When}\;\left[S\right]}_{T}\ll K_{m},V}_{0}=\frac{k_{\text{cat}}}{K_{m}}{\;\left[S\right]}_{T}{\;\left[E\right]}_{T}$$

SQOR uses three substrates: H_2_S, a sulfur acceptor (Acc^−^), and CoQ (Fig. 1*A*). At saturating concentrations of H_2_S and CoQ, the *k*_cat_/*K*_m_^Acc-^ represents the apparent rate constant for the reaction between the sulfur acceptor and the Cys_379_SSH of the bis-persulfidated SQOR, and its pH-dependency reports on the p*K*_a_ of both free species. Although the pH dependence of human SQOR activity has been reported (25), the pH dependence of *k*_cat_/*K*_m_ is unavailable. The steady-state rate of SQOR was determined by monitoring the reduction of coenzyme Q_1_ (CoQ_1_) at 278 nm at varying pH at 25 °C. Sulfite or cyanide was used as the sulfur acceptor, and saturating concentrations of H_2_S (150 μM) and CoQ_1_ (69 μM, *K*_m_^CoQ1^ = 19 μM, pH 7.5 (25)) were used. Note that this concentration of H_2_S is saturating for the first step of the catalysis (Fig. 1*A*, reaction *a*), *K*_m_^H2S (Donor)^ = 13 μM at pH 7.5 (25), but not for the second step with H_2_S acting as the sulfur acceptor (Fig. 1*A*, reaction *b*), *K*_m_^H2S (Acceptor)^ = 350 μM at pH 7.4 (27). The reaction between the persulfidated enzyme and H_2_S (*k*_cat_/*K*_m_^H2S (Acceptor)^ = 1.8 × 10^5^ M^−1^ s^−1^, pH 7.4) is negligible in the presence of sulfite (*k*_cat_/*K*_m_^sulfite^ = 2.0 × 10^6^ M^−1^ s^−1^, pH 7.4) but comparable to the reaction with cyanide (*k*_cat_/*K*_m_^cyanide^ = 5.1 × 10^5^ M^−1^ s^−1^, pH 8.5) (25, 27). Therefore, in the experiments with cyanide, the reactions were started with cyanide instead of SQOR so that the contribution of SQOR with H_2_S alone could be subtracted.

**Figure 3:**
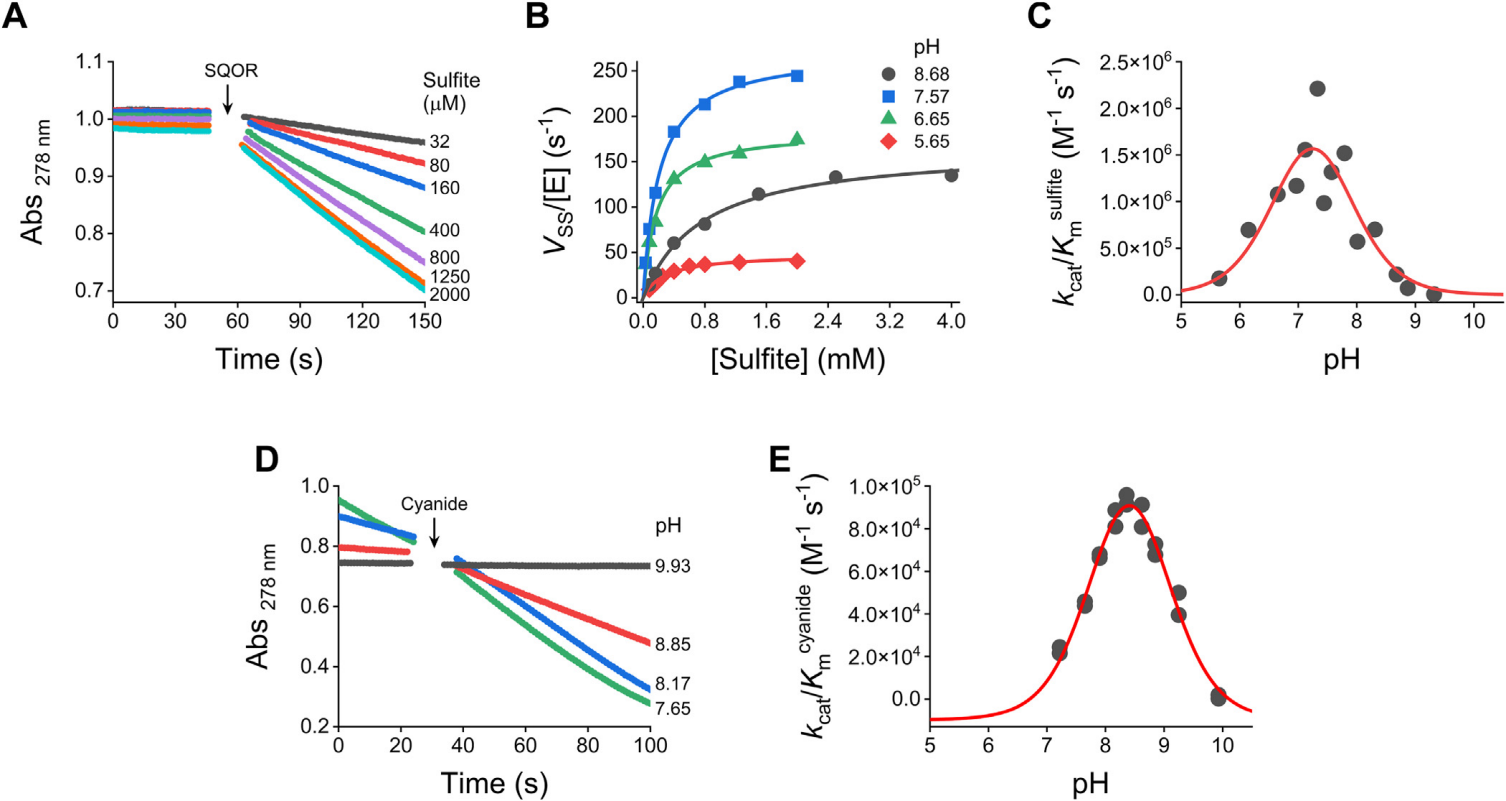
**pH-dependence of SQOR activity.** The steady-state rate of reduction of CoQ_1_ was followed by the decrease in absorbance at 278 nm. The assays included 69 μM CoQ_1_, 0.03% DHPC, 0.06 mg/ml BSA, 150 μM H_2_S, and variable concentrations of sulfite or cyanide in MES/Tris/ethanolamine buffer (pH 5.65–9.93, 25 °C). *A*, the reactions with sulfite (0.01–8 mM) were started by the addition of 1 nM SQOR. Representative absorbance kinetic traces of CoQ_1_ reduction at pH 7.57. The steady-state rates were calculated from the linear fits to the data obtained 15 to 30 s after SQOR was added (subtracting the slopes before addition of SQOR). *B*, SQOR activity *versus* sulfite concentration at different pH values. Representative experiments at pH 8.68 (*black circles*), 7.57 (*blue squares*), 6.65 (*green triangles*), and 5.65 (*red diamonds*). Michaelis-Menten hyperbolas were fitted and yielded the kinetic parameters *K*_m_^sulfite^ (Fig. S1*A*), *k*_cat_^sulfite^ (Fig. S1*B*), and *k*_cat_/*K*_m_^sulfite^ for each pH. *C*, pH-dependence of *k*_cat_/*K*_m_^sulfite^. Equation 3 was fitted to the data obtaining two p*K*_a_s, 6.8 ± 0.5 for the deprotonated species and 7.7 ± 0.4 for the protonated one. A pH-independent *k*_cat_/*K*_m_^sulfite^ of 2.9 ± 0.2 × 10^6^ M^−1^ s^−1^ was obtained for the reaction of sulfite with the persulfidated SQOR. *D*, the reactions with cyanide (90 μM) contained 50 nM SQOR and were initiated by the addition of cyanide. Representative time courses at different pHs. To calculate the steady-state rates, the slopes in the absence of cyanide were subtracted from those obtained 4 to 20 s after the addition of cyanide. The *k*_cat_/*K*_m_^cyanide^ at each pH was calculated using Equation 2. *E*, pH-dependence of *k*_cat_/*K*_m_^cyanide^. Equation 3 plus an offset was fitted to the data, yielding a p*K*_a_ of 8.9 ± 0.2 for the deprotonated species, a p*K*_a_ of 7.9 ± 0.1 for the protonated species, an offset of (−10 ± 5) × 10^3^ M^−1^ s^−1^, and a pH-independent *k*_cat_/*K*_m_^cyanide^ of 1.5 ± 0.8 × 10^6^ M^−1^ s^−1^ for the reaction of cyanide with the persulfidated SQOR. Values are parameters ± standard errors of the fits. DHPC, 1,2-diheptanoyl-sn-glycero-3-phosphocholine; SQOR, sulfide quinone oxidoreductase.

In experiments with sulfite, steady-state time courses with varying concentrations of sulfite (0.01–8 mM) were recorded, and linear functions were fitted to the first 15 to 40 s following SQOR addition (Fig. 3*A*). The slope before SQOR addition was subtracted to correct for the background nonenzymatic reduction of CoQ_1_, which was minimal. From the hyperbolic fit of the activity *versus* sulfite concentration plot (Fig. 3*B*), the kinetic parameters *k*_cat_^sulfite^ and *K*_m_^sulfite^ were obtained, and the *k*_cat_/*K*_m_^sulfite^ for each pH were calculated. As expected, when the *k*_cat_/*K*_m_^sulfite^ were plotted against pH, a bell-shaped profile was obtained (Fig. 3*C*), consistent with a reaction between a deprotonated species and a protonated one. An equation with two p*K*_a_ was fitted to the data (Equation 3), assuming that deprotonated sulfite and protonated SQOR persulfide were the reacting species. From this analysis, a p*K*_a_ value of 6.8 ± 0.5 was obtained for sulfite, which is remarkably consistent with the reported value of 6.91 (34). The p*K*_a_ attributed to the protonated persulfide on SQOR was 7.7 ± 0.4. Since sulfite reacts with the persulfide on Cys_379_, it can be inferred that this p*K*_a_ corresponds to Cys_379_SSH. Although the assignment to another catalytic residue present in the bis-persulfidated SQOR cannot be excluded, it is unlikely to correspond to the persulfide formed in Cys_201_, since this persulfide is engaged with the FAD cofactor in the formation of the CT complex. The bell-shaped fit also revealed a pH-independent *k*_cat_/*K*_m_^sulfite^ of (2.9 ± 0.2) × 10^6^ M^−1^ s^−1^. The maximum *k*_cat_/*K*_m_^sulfite^ was observed at pH 7.25. Both *k*_cat_^sulfite^ and *K*_m_^sulfite^ varied with pH (Fig. S1, *A* and *B*). Of note, the results obtained in these experiments are consistent with previous measurements in potassium phosphate at pH 7.4, which showed a *k*_cat_/*K*_m_^sulfite^ of 2.1 × 10^6^ M^−1^ s^−1^ (25 °C) and a second-order rate constant between the enzyme CT complex and sulfite of 3.9 × 10^5^ M^−1^ s^−1^ (4 °C) (25, 27).

(Equation 3) $$k_{\text{pH}}=k_{\text{ind}}\left(\frac{K_{a}^{{\;\text{Acc}}^{-}}}{K_{a}^{{\;\text{Acc}}^{-}}+\left[H^{+}\right]}\right)\left(\frac{\left[H^{+}\right]}{K_{a}^{\;\text{RSSH}}+\left[H^{+}\right]}\right)$$

Kinetic traces with cyanide (Fig. 3*D*) were recorded at varying pH using 90 μM cyanide, which is lower than the *K*_m_^cyanide^ (650 μM at pH 8.5 (25)). The steady-state rates were obtained from linear fits to the data during the first 4 to 20 s following cyanide addition, and the slopes before cyanide addition were subtracted. The *k*_cat_/*K*_m_^cyanide^ at each pH was calculated according to Equation 2 using the enzyme (50 nM) and cyanide (90 μM) initial concentrations. The pH-dependence of *k*_cat_/*K*_m_^cyanide^ exhibited bell-shaped behavior and Equation 3 was fitted to the data (Fig. 3*E*). A p*K*_a_ of 8.9 ± 0.2 was obtained for the deprotonated species, consistent with the p*K*_a_ of 8.97 expected for HCN under these conditions (*I* = 0.15 M, 25 °C) (35). It should be noted that the p*K*_a_ of HCN changes considerably with temperature and ionic strength (*I*); the values often cited, 9.21 and 9.36, correspond to *I* = 0 at 25 and 20 °C, respectively (35). For the persulfidated enzyme, a p*K*_a_ of 7.9 ± 0.1 was obtained, in excellent agreement with the results with sulfite (7.7 ± 0.4). Furthermore, the pH-independent *k*_cat_/K_m_^cyanide^ was (1.5 ± 0.8) × 10^6^ M^−1^ s^−1^. Note that only a small fraction of the pH-independent constant was observed (∼7% at pH 8.4) (Fig. 3*E*), since cyanide, which needs to be deprotonated, has a higher p*K*_a_ than the persulfidated enzyme that appears to be protonated for catalysis.

Controls confirmed that the low activities seen at the extreme pH values with sulfite and cyanide were due to reversible changes in protonation states instead of irreversible denaturation of SQOR (Fig. S1*B*). Additionally, the concentrations of H_2_S and CoQ_1_ used were observed to be saturating at all pHs (Fig. S1*C*).

Attempts to use GSH as sulfur acceptor to assess *k*_cat_/*K*_m_^GSH^ at different pHs were unsuccessful. When relatively low concentrations of GSH were used, SQOR reacted with H_2_S as acceptor (*k*_cat_/*K*_m_^H2S (Acceptor)^ = 1.8 × 10^5^ M^−1^ s^−1^, pH 7.4) rather than with GSH (*k*_cat_/*K*_m_^GSH^ = 1.1 × 10^4^ M^−1^ s^−1^, pH 7.4) (27). On the other hand, construction of Michaelis–Menten hyperbolas required high concentrations of GSH (*K*_m_^GSH^ = 8 mM, pH 7.4 (27)), which altered the pH of the reaction mixtures, and could not be compensated without changing the ionic strength.

Note that in the p*K*_a_ determination experiments, chloride (Cl^−^) was avoided since it was found to be a reversible competitive inhibitor of SQOR. At neutral pH with sulfite as sulfur acceptor, relatively similar apparent *k*_cat_^sulfite^ were achieved in the presence and absence of 120 mM chloride, but the apparent *K*_m_^sulfite^ increased ∼ 20-fold (estimated *K*_i_ for chloride was ∼ 7.4 mM) (Fig. S1*D*).

The pH experiments with SQOR, summarized in Table 2, suggest that the Cys_379_ persulfide reacts in the protonated state with the nucleophilic sulfur acceptor and has a p*K*_a_ of 7.8 ± 0.2.

**Table 2:** p*K*_a_ and rate constants for SQOR and TST, at *I* = 0.15 and 25 °C

Enzyme form	p*K*_a_	pH-independent rate constant (M^−1^ s^−1^)		
		Sulfite	Cyanide	Thiosulfate
SQOR persulfide	7.8 ± 0.2^a^	(2.9 ± 0.2) × 10^6^	(1.5 ± 0.8) × 10^6^	No reaction
TST persulfide	9.38 ± 0.04^a^	(2.5 ± 0.1) × 10^5^	(1.0 ± 0.1) × 10^7^	No reaction^b^
TST thiol	6.5 ± 0.1	No reaction	No reaction	∼ 6 × 10^4^

a Values represent mean ± propagated error of the results obtained with sulfite and cyanide.

b Thiosulfate is not a substrate for TST persulfide, it acts as an inhibitor.

### Thiosulfate sulfurtransferase

**Figure 4:**
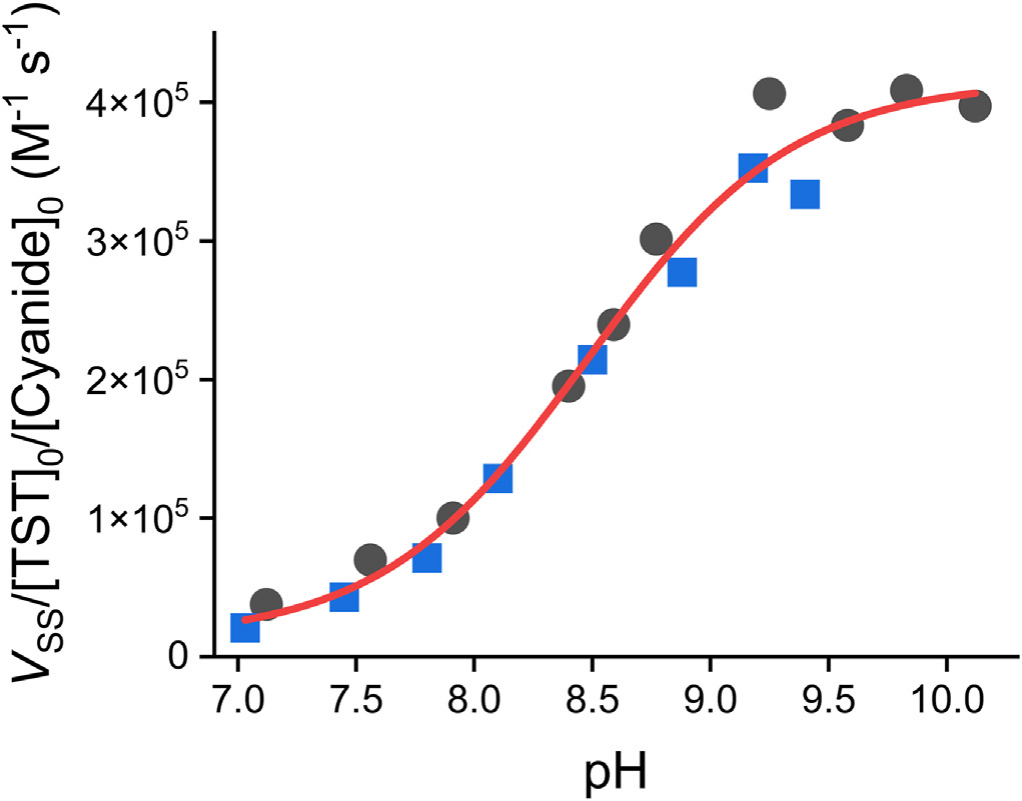
**pH-dependence of TST activity.** The steady-state activity of TST (5–100 nM) was measured by the formation of thiocyanate in the presence of thiosulfate (300 mM) and cyanide (300 μM, lower than *K*_m_^cyanide^) in ACES/Tris/ethanolamine buffer in the pH range of 7.03 to 10.12 and 25 °C. A sigmoidal function was fitted to the data of two independent experiments (*black circles* and *blue squares*) and yielded an apparent p*K*_a_ of 8.47 ± 0.06 and a maximum apparent rate constant of (4.0 ± 0.1) × 10^5^ M^−1^ s^−1^ (parameters ± standard errors of the fit). TST, thiosulfate sulfurtransferase.

We first measured TST activity by monitoring the formation of thiocyanate at varying pH at 25 °C. Saturating concentrations of thiosulfate (300 mM, *K*_m_^thiosulfate^ = 18–45 mM (30, 31)) and cyanide concentrations lower than the *K*_m_^cyanide^ (300 μM, *K*_m_^cyanide^ = 1.8–2.8 mM (31), and this work) were used so that the global rates would be limited by the last step of the catalytic mechanism (Fig. 1*B*, reaction *f*), which involves the rate constant of the reaction between the persulfidated enzyme and cyanide, *k*_cat_/*K*_m_^cyanide^. The spontaneous reaction between the substrates at different pH was found to be negligible under our conditions, in accordance with reported data (36). A sigmoidal increase in activity with pH was observed, with an apparent p*K*_a_ of 8.47 ± 0.06 and a maximum apparent rate constant of (4.0 ± 0.1) × 10^5^ M^−1^ s^−1^ at alkaline pH (Fig. 4). Control experiments excluded irreversible enzyme inactivation (Fig. S2*A*). Additionally, controls performed at the most acidic and alkaline pH confirmed that the concentration of thiosulfate was saturating and that the concentration of cyanide was below *K*_m_^cyanide^ (Fig. S2*B*). Of note, the buffer system affected the enzyme activity; using a 300 mM Tris buffer with 120 mM NaCl, the activity was <40% of that obtained with the ACES/Tris/ethanolamine buffer at the same pH, which contained 15.6 mM Tris and 120 mM NaCl.

Stopped-flow kinetic studies were performed on the isolated half-reactions between TST persulfide and sulfur acceptors. Preformed stocks of TST persulfide were used to monitor its reaction with sulfite or cyanide at varying pH at 25 °C. The reactions were followed by the changes in the intrinsic fluorescence of TST, taking advantage of the higher fluorescence in the thiol *versus* the persulfidated state, as reported for the bovine enzyme (37). The decrease in fluorescence appears to be due to energy transfer involving tryptophans and the persulfide, without major folding rearrangements (38), hence it likely reports on persulfide formation.

**Figure 5:**
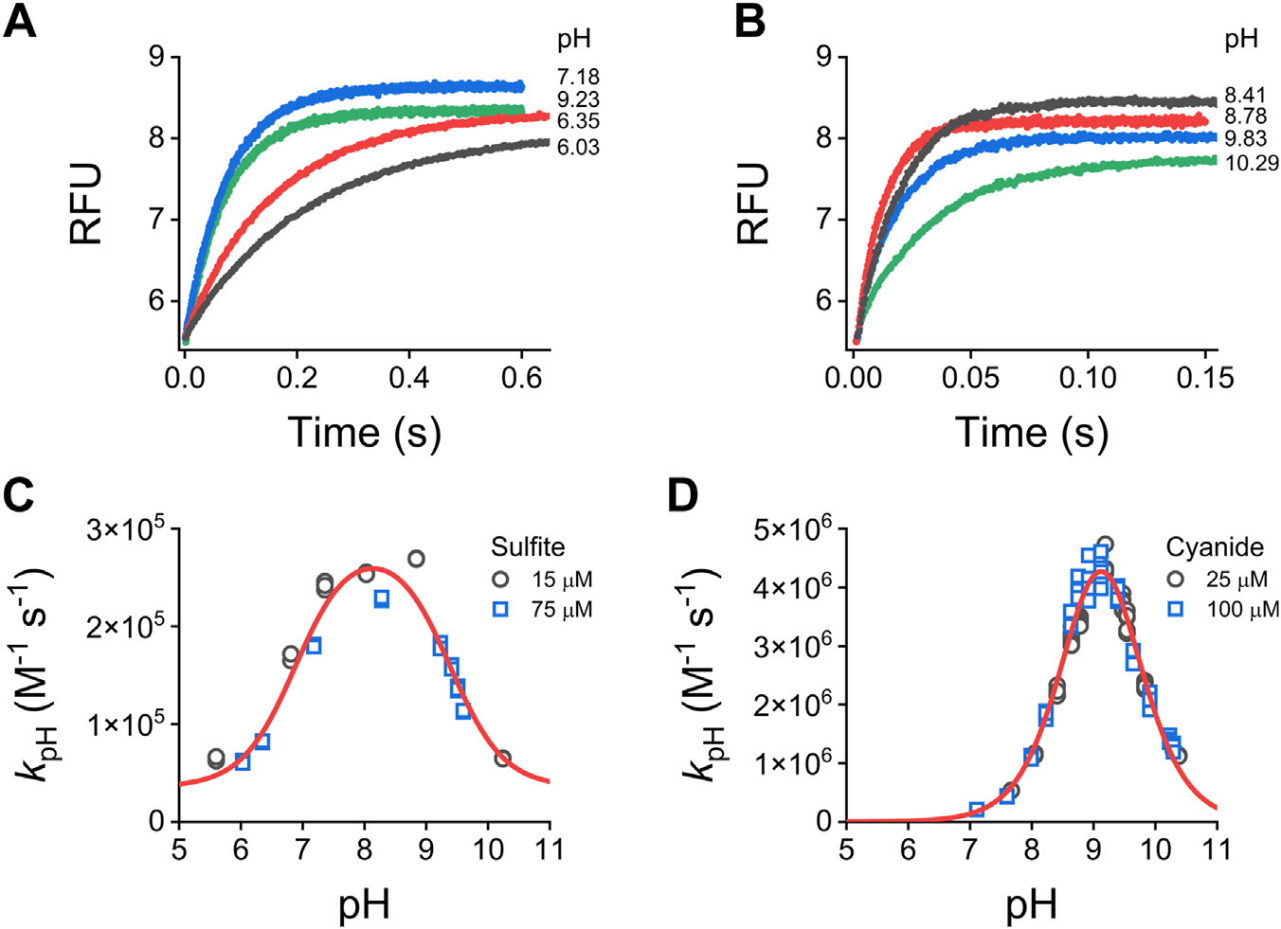
**Stopped-flow kinetics of TST persulfide with sulfur acceptors.***A* and *B*, representative fluorescence time courses (λ_ex_ = 295 nm, US 360 nm bandpass filter) of TST persulfide (0.8–1.0 μM) exposed to 75 μM sulfite (*A*) or 25 μM cyanide (*B*), in ACES/Tris/ethanolamine buffer (pH 5.60–10.38, 25 °C). Single exponential functions were fitted and the *k*_obs_ was divided by the concentration of sulfite or cyanide to give the corresponding *k*_pH_. *C* and *D*, pH-dependence of *k*_pH_. Bell-shaped functions (Equation 3 or Equation 3 plus an offset) were fitted to the data, yielding p*K*_a_ values of 6.89 ± 0.09 (deprotonated species) and 9.38 ± 0.07 (protonated species), an offset of (3.5 ± 0.9) × 10^4^ M^−1^ s^−1^, and a *k*_ind_ of (2.5 ± 0.1) × 10^5^ M^−1^ s^−1^ for the reaction with sulfite (*C*) and p*K*_a_ values of 8.87 ± 0.06 (deprotonated species) and 9.37 ± 0.05 (protonated species) and a *k*_ind_ of (1.0 ± 0.1) × 10^7^ M^−1^ s^−1^ for the reaction with cyanide (parameters ± standard errors of the fits). TST, thiosulfate sulfurtransferase.

TST persulfide was exposed to a pseudo-first order excess of sulfite (15 and 75 μM) or cyanide (25 and 100 μM), and the increases in the intrinsic fluorescence were recorded (Fig. 5, *A* and *B*). Single exponential functions were fitted to the time courses. The *k*_obs_ values were divided by the sulfite or cyanide concentration and the second order rate constants, *k*_pH_, were determined at each pH. The *k*_pH_ for both sulfur acceptors showed bell-shape profiles, and the Equation 3 was fitted (Fig. 5, *C* and *D*). In the case of sulfite, the fit yielded a p*K*_a_ of 6.89 ± 0.09 for the deprotonated species, consistent with sulfite (p*K*_a_ = 6.91 (34)), and a p*K*_a_ of 9.38 ± 0.07 for the protonated species. The pH-independent rate constant, *k*_ind_, was (2.5 ± 0.1) × 10^5^ M^−1^ s^−1^ (Fig. 5*C*). With cyanide on the other hand, the fit gave a p*K*_a_ of 8.87 ± 0.06 for the deprotonated species, in agreement with cyanide (p*K*_a_ = 8.97 (35)), and a p*K*_a_ of 9.37 ± 0.05 for the protonated species. The *k*_ind_ was (1.0 ± 0.1) × 10^7^ M^−1^ s^−1^ (Fig. 5*D*). Notably, the p*K*_a_ value for the protonated species was the same with both sulfur acceptors (9.37 and 9.38) and thus, it can be attributed to persulfidated TST. In the case of the bovine enzyme, a previous report suggested p*K*_a_s of 5.9 and 9.4 for the persulfide derivative (30); our results are in good agreement with the alkaline value.

**Figure 6:**
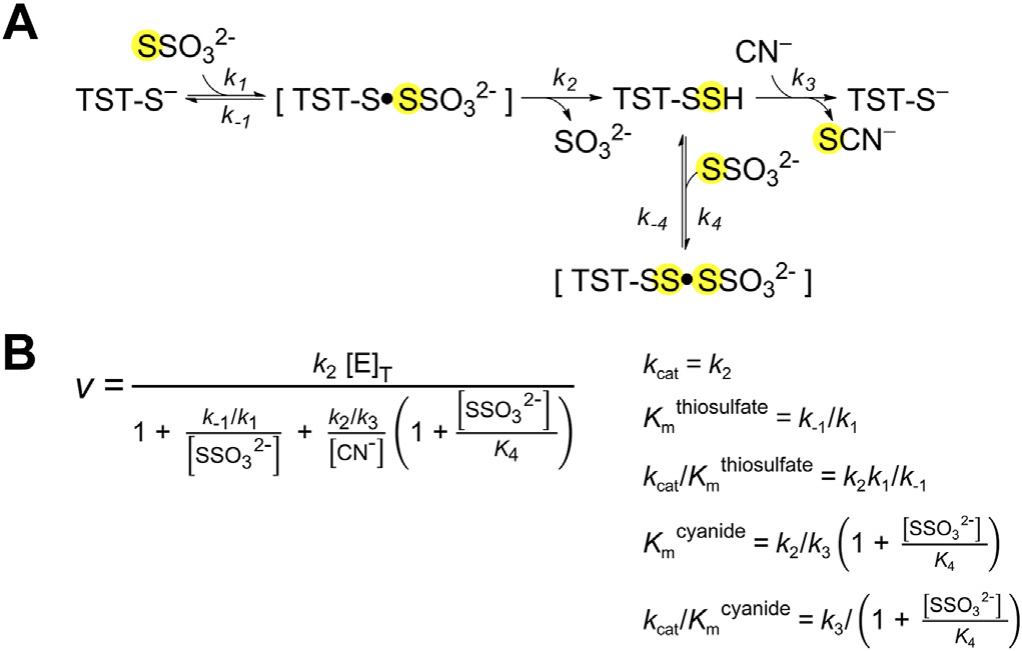
**TST mechanism and inhibition by thiosulfate.***A*, TST catalytic mechanism using thiosulfate and cyanide as substrates and depicting inhibition by high concentrations of thiosulfate (30, 31). *B*, steady-state rate equation assuming fast equilibrium for thiosulfate binding and steady-state for the persulfidated enzyme. TST, thiosulfate sulfurtransferase.

The *k*_ind_ for the reaction of TST persulfide with cyanide, (1.0 ± 0.1) × 10^7^ M^−1^ s^−1^, is 25-fold higher than the maximum rate constant obtained from the activity measurements with 300 mM thiosulfate and 300 μM cyanide, 4.0 × 10^5^ M^−1^ s^−1^ (Fig. 4). The lower value obtained in the activity measurements can be explained by inhibition at high concentrations of thiosulfate which leads to the formation of a dead-end complex between the persulfidated enzyme and thiosulfate (30, 31) (Fig. 6*A*). In fact, with 312 μM cyanide, 30 mM thiosulfate can already cause inhibition (31). Therefore, the steady-state rates measured herein were strongly affected by thiosulfate inhibition (Fig. 6*B*). The extent to which *k*_cat_/*K*_m_^cyanide^ (*k*_3_ in Fig. 6*A*) was affected by thiosulfate inhibition depends on the thiosulfate concentration as well as on the dissociation constant of the dead-end complex *K*_4_, defined as *k*_-4_/*k*_4_ (Fig. 6*A*); the observed *k*_cat_/*K*_m_^cyanide^ is given by *k*_3_/(1+[thiosulfate]/*K*_4_) (Fig. 6*B*). Thus, the pH-dependency of *K*_4_ can influence the experiment, complicating the interpretation. Human TST can be activated by cyanide at high millimolar concentrations (31), which is unlikely to affect our results. Of note, the values of *k*_cat_/*K*_m_^cyanide^ reported for the bovine and human enzymes are 6.0 × 10^5^ M^−1^ s^−1^ (pH 8.7, 40 °C) and 9.5 × 10^4^ M^−1^ s^−1^ (pH 8.5, 0 °C), respectively, under noninhibited conditions (30, 31). At pH 8.5, our stopped-flow estimation of the rate constant between the persulfidated enzyme and cyanide is higher, of 2.7 × 10^6^ M^−1^ s^−1^ (25 °C), and deserves further exploration. It is worth noting that a reaction between TST persulfide and mBrB was explored at varying pH to allow comparison with LMW persulfides. However, no such reaction was detected under our conditions.

**Figure 7:**
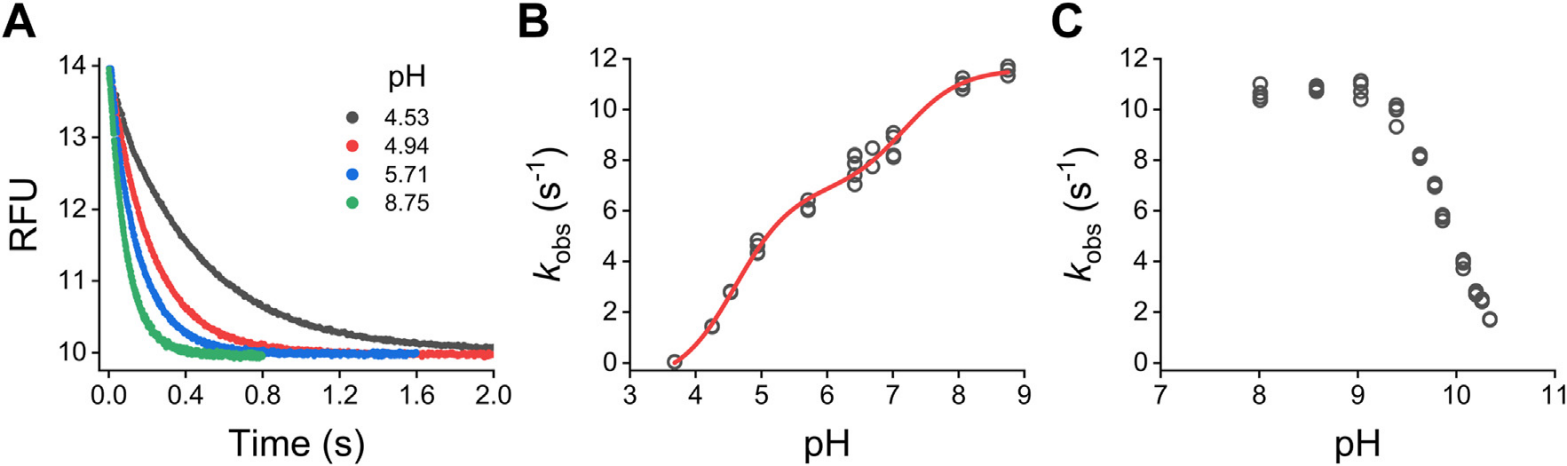
**Stopped-flow kinetics of TST thiol with thiosulfate.***A*, representative fluorescence time courses (λ_ex_ = 295 nm, US 360 nm bandpass filter) of TST thiol (0.9 μM) exposed to 200 μM thiosulfate in acetic/MES/Tris buffer (pH 3.68–8.75, 25 °C). Single or double exponential functions were fitted to the data. *B*, pH-dependence of *k*_obs_ for a representative experiment. For the double exponential fits, the smaller *k*_obs_ values, which corresponded to the larger amplitude, were used. Using data from three independent experiments, a two-p*K*_a_ sigmoidal function was fitted yielding p*K*_a_ values of 4.6 ± 0.1 and 6.5 ± 0.1 and maximal *k*_obs_ of 5.9 ± 0.6 and 11.5 ± 0.3 s^−1^, respectively (parameters ± standard errors of the fit). *C*, pH-dependence of the *k*_obs_ at alkaline pH values. The reactions were performed as described but in ACES/Tris/ethanolamine buffer (pH 6.68–10.34, 25 °C), and a single exponential plus straight line function was fitted to the data to obtain the *k*_obs_. Representative of two independent experiments, five replicates each. TST, thiosulfate sulfurtransferase.

The kinetics of the reaction of TST thiol with pseudo-first order concentrations of thiosulfate (200 μM) was investigated in a pH range of 3.68 to 8.75 and 25 °C. Since thiosulfate does not accept or release protons within the pH range studied, the pH-dependence of the reaction rate should reveal the p*K*_a_ of the TST thiol. The decrease in the intrinsic fluorescence of TST due to persulfide formation was recorded (Fig. 7*A*). The kinetic traces followed single or double exponential functions, which is consistent with the formation of TST persulfide in two steps with a non-covalent intermediate (Fig. 6*A*). The smaller *k*_obs_, which corresponded to the larger amplitude, was found to increase with pH (Fig. 7*B*), consistent with the thiolate enzyme, rather than the protonated thiol, being the species reacting with thiosulfate. The data followed a two-p*K*_a_ sigmoidal function, indicating the presence of two reacting species with p*K*_a_ values of 4.6 ± 0.1 and 6.5 ± 0.1, and second-order rate constants of ∼ 3 × 10^4^ and ∼ 6 × 10^4^ M^−1^ s^−1^ (estimated by dividing the maximal *k*_obs_ obtained from the fit, 5.9 and 11.5 s^−1^, respectively, by thiosulfate concentration). This result suggests that the ionization state of a neighboring residue affects the p*K*_a_ of the thiol. Our estimated p*K*_a_ of 6.5 ± 0.1 can be compared to previous reports; a p*K*_a_ of 7.8 was reported for the thiol alone in bovine TST (39), while p*K*_a_ values of 6.5 and 6.75 to 7.05 were reported for the thiol in complex with thiosulfate and with substrate analogs, respectively (30, 39). In human 3-mercaptopyruvate sulfurtransferase, the thiol was reported to have a p*K*_a_ of 5.2 (40). Regarding the rate constants for the reaction of TST thiol with thiosulfate, our value (∼ 6 × 10^4^ M^−1^ s^−1^) can be compared to the value of *k*_cat_/*K*_m_^thiosulfate^ of 1.7 × 10^4^ M^−1^ s^−1^ (40 °C, pH 8.7) reported for the bovine enzyme (30, 31). The *k*_cat_/*K*_m_^thiosulfate^ parameter represents an algebraic combination of the kinetic constants corresponding to the first and second steps of the mechanism, *k*_2_*k*_1_/*k*_-1_ (Fig. 6*B*). Last, the kinetic analysis was extended to higher pH values, and a decrease in *k*_obs_ with a p*K*_a_ of ∼ 10 was observed (Fig. 7*C*). This p*K*_a_ is comparable to the value of 9.9 reported for an increase in *K*_m_^thiosulfate^ (*i.e.*, *k*_-1_/*k*_1_) at alkaline pH in the bovine enzyme (30) and likely reflects the effect of deprotonation of one or more residues other than the thiol, decreasing the reaction between thiosulfate and the thiolate.

The results with TST are summarized in Table 2. Taken together, they reveal that the reaction of persulfidated TST with sulfur acceptors is dependent on a protonated residue with a p*K*_a_ of 9.38 ± 0.04. In contrast, the TST thiol reacts with thiosulfate in the anionic state, with a p*K*_a_ of 6.5 ± 0.1.

## Discussion

In this study, the acidity and rate constants of the reaction between mBrB and a series of LMW alkyl persulfides were measured (Table 1). When analyzing the p*K*_a_ and *k*_ind_ of the persulfides, it is important to consider the ionization state of other groups. In the case of β-MESSH, in which the persulfide is the only ionizable group in the working pH range, the assignment of the p*K*_a_ and *k*_ind_ is straightforward. In the case of CystSSH, the p*K*_a_ and *k*_ind_ can be assigned to the persulfide in the species containing ammonium (as drawn in Table 1), since the ammonium has a p*K*_a_ of 9.55 in the analogous thiol (41), favoring protonation of the amino group at the working pH. In the case of amino acid–derived persulfides, the p*K*_a_ and *k*_ind_ determined with mBrB likely correspond to the forms with ionized carboxylate and ammonium (as drawn in Table 1 for CysSSH and HcySSH), given that the analogous thiols have p*K*_a_ values of ⁓2 for the carboxylic acid and >9 for the ammonium group (16), distant from the observed p*K*_a_s. In the case of GSSH, although the glycyl carboxylic acid has p*K*_a_ values of 3.12 and 3.36 for the positive and neutral variants of GSH, respectively (42), we observed only one p*K*_a_ of 5.45 for the persulfide (13), suggesting that it corresponds to the form with two anionic carboxylates and ammonium. Last, in the case of CysOMeSSH (Fig. 2*G*), the two sets of p*K*_a_ and *k*_ind_ obtained likely correspond to the microscopic constants of the ammonium- and the amino-persulfide forms. The observation of microscopic constants for CysOMeSSH is probably related to proximity between the p*K*_a_s of the persulfide and the ammonium groups, since the latter have values of 6.88 and 8.32 in CysOMeSH and CysOMeS^−^, respectively (43). Microscopic constants have also been observed for reactions of mBrB with several thiols (16, 44).

**Figure 8:**
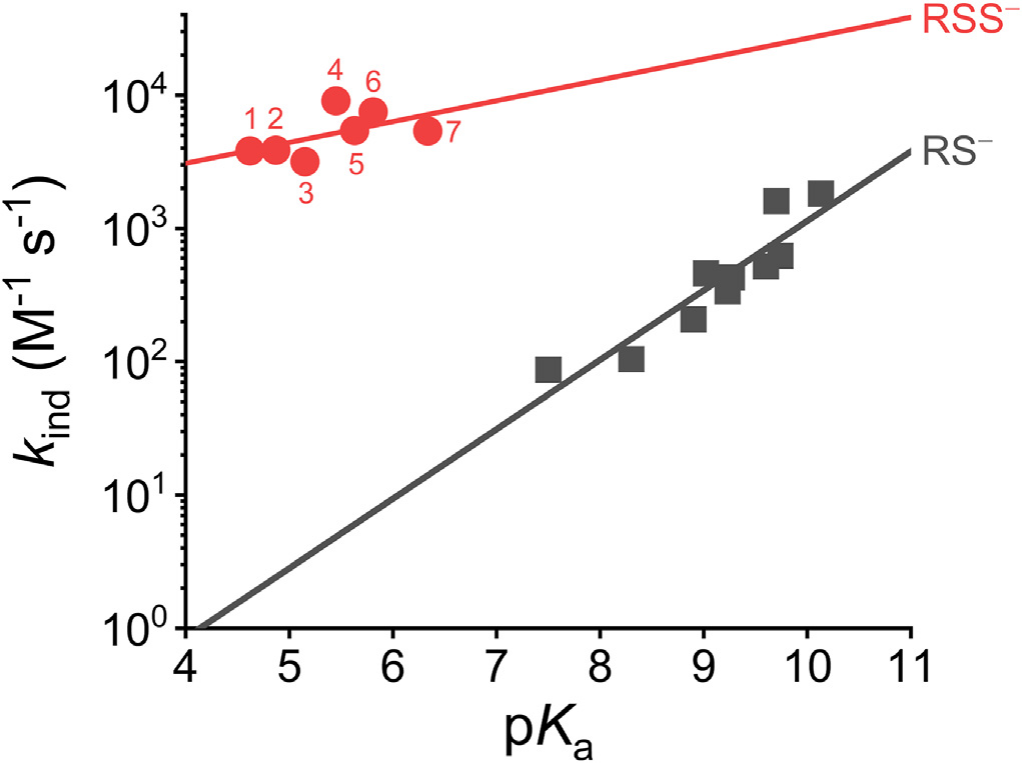
**Comparison of the reactivity of LMW persulfide anions and thiolates with mBrB.** Brønsted plot exhibiting pH-independent rate constants (in logarithmic scale) *versus* p*K*_a_ for the reactions of persulfide anions or thiolates with mBrB (*I* = 0.15 and 25 °C). *Red* circles: CysOMeSS^−^(NH_3_^+^) (1), CystSS^−^ (2), CysSS^−^ (3), GSS^−^ (4), HcySS^−^ (5), β-MESS^−^ (6), and CysOMeSS^−^(NH_2_) (7); *β*_nuc_ = 0.2 ± 0.1 (R^2^ 0.31). The values are depicted in Table 1. *Black squares*: reported data for LMW thiolates; *β*_nuc_ = 0.52 ± 0.08 (R^2^ = 0.85) (16). LMW, low molecular weight; mBrB, monobromobimane.

Remarkably, the persulfides studied presented similar acidity, with p*K*_a_ values around 5.4. Despite the similarity, when sorted by p*K*_a_, the resulting order was roughly the same as that of the analogous thiols (Table 1). These low p*K*_a_ values confirm that LMW persulfides exist predominately in the anionic state at physiological pH. Furthermore, the pH-independent rate constants (*k*_ind_) for the reaction between the different nucleophilic persulfides with mBrB were similar, ∼ 10^3^ M^−1^ s^−1^ (Table 1). In contrast, for thiols, both the acidity and the *k*_ind_ values are spread over a larger range. These results can be visualized in the Brønsted plot shown in Figure 8, which depicts the *k*_ind_ in logarithmic scale as a function of p*K*_a_ for both persulfides and thiols. Clearly, our results demonstrate that substituents exert a limited effect on the p*K*_a_ of persulfides in comparison to thiols, likely because of the presence of an additional sulfur, which increases the distance between the substituents and the outer sulfur.

The LMW persulfides studied showed higher reactivity with mBrB than the analogous thiols (Table 1). Increased nucleophilicity has been observed for aromatic persulfides *versus* thiolates (45), and polysulfides (HS_n_^−^) *versus* HS^−^ (46), although not for zinc polysulfide *versus* zinc thiolate compounds (47). In addition, higher reactivity of persulfides has been observed in proteins such as human serum albumin and the peroxiredoxin AhpE when reacting with unspecific electrophiles (5, 48). Computational studies also support a higher reactivity for HSS^−^ *versus* HS^−^ or RSS^−^ *versus* RS^−^ (5, 13, 49), although the extent of acceleration has been questioned (50).

The nucleophilic reactivities of LMW persulfides with mBrB can also be compared with the reactivities of thiols with similar basicity. This comparison can be visualized by deviations in the *y*-axis of Brønsted plots. The positive deviation of persulfides with respect to the trend followed by the thiolates (Fig. 8) indicates that persulfides have higher rate constants than putative thiols with the same p*K*_a_, constituting evidence for the alpha effect in the reaction of alkyl persulfides with mBrB (*i.e.,* the increased reactivity of a nucleophile that has an adjacent atom with high electron density in comparison to a reference nucleophile with similar p*K*_a_ (17, 18)). The magnitude of the alpha effect depends not only on the nucleophile but also on the electrophile. For example, GSS^−^ reacts 1670-fold faster than a thiolate with similar basicity with mBrB but only 3.2-fold faster with hydrogen peroxide (13). The origin of the alpha effect remains elusive; possible explanations include transition state stabilization, ground state destabilization, and solvation differences. With persulfides, an attractive hypothesis is the increased stabilization of the biradical character of the transition state (51). In this regard, the free radicals derived from the one-electron oxidation of persulfides are more stable than those derived from thiols (14, 15).

The slope of the Brønsted plot is called β_nuc_. Although the estimation of the β_nuc_ for persulfides was subject to high uncertainty due to the clustering of the persulfide values in the Brønsted plot, its value was lower than the β_nuc_ for thiols (β_nuc_^RSSH^ = 0.2 ± 0.1, β_nuc_^RSH^ = 0.52 ± 0.08) (Fig. 8). This is reminiscent of the β_nuc_ difference between oximates (alpha nucleophiles) and phenoxides (52). Additional studies are needed to understand the basis of the differences in β_nuc_ between persulfides and thiols.

**Figure 9:**
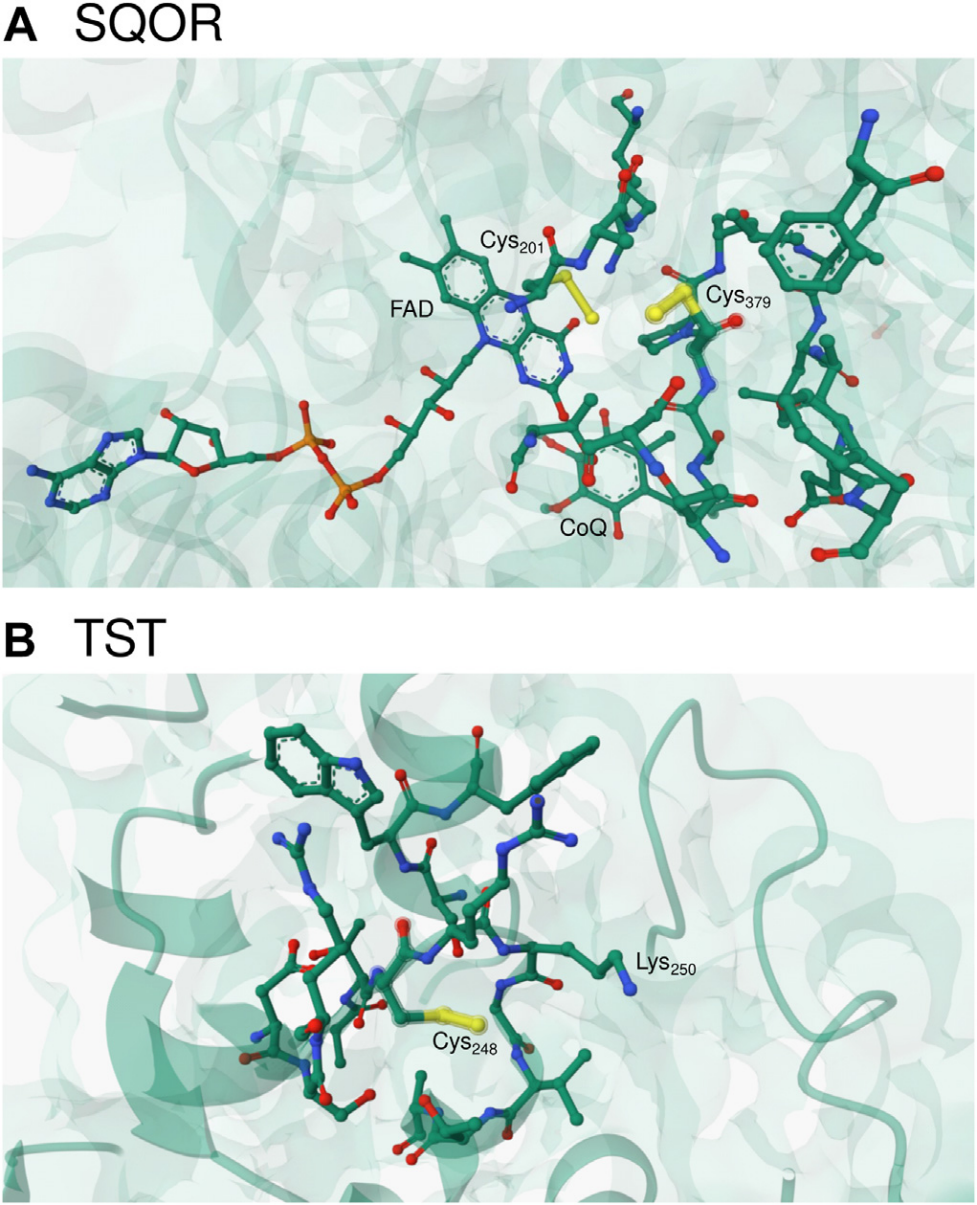
**Persulfides in SQOR and TST.***A*, close-up of the bis-persulfide in the SQOR structure (PDB 6OIB). Residues within 5 Å from Cys_379_SSH, the FAD cofactor, and the CoQ substrate are depicted in *sticks*. *B*, close-up of the persulfide in the bovine TST structure (PDB 1RHD). Residues within 5 Å from Cys_248_ are depicted in *sticks*. Figures were constructed with Mol∗ (65). SQOR, sulfide quinone oxidoreductase; TST, thiosulfate sulfurtransferase.

The data on persulfidated SQOR and TST underscore the scope for modulating reactivity by the protein scaffold. The pH-dependence of the SQOR steady-state rate under conditions that report on the persulfidated enzyme suggested that the persulfide on Cys_379_ is in the protonated state for reaction with the sulfur acceptors and has a p*K*_a_ of 7.8 ± 0.2. This increase in SQOR p*K*_a_ in comparison to a LMW persulfide (p*K*_a_ ∼ 5.4) favors a larger fraction of protonated persulfide on Cys_379_, which would promote the electrophilic character of the outer sulfur and avoid repulsion with the negative charge of either sulfite or cyanide. The crystal structure of bis-persulfidated SQOR (PDB 6OIB) shows that the Cys_379_SSH is located in an electropositive cavity that is exposed to solvent (21). No clear hydrogen-bonding partners for the outer sulfur are seen, and the proximity to the anionic persulfide located in Cys_201_ would promote an uncharged Cys_379_SSH (Fig. 9).

A serendipitous finding of our work was that SQOR is inhibited by chloride, which competes with sulfite (*K*_i_ ∼ 7.4 mM). Since the chloride concentration in the mitochondrial matrix is estimated to be ∼ 4.2 mM (53), SQOR might be partially inhibited by chloride *in vivo*.

Substrate inhibition of TST by thiosulfate is unlikely to have physiological relevance, since intracellular thiosulfate levels are estimated to be 5 to 20 μM (54, 55), while inhibition of TST is achieved at > 30 mM thiosulfate (31). Nevertheless, this inhibition affected our steady-state kinetic results with TST, complicating their interpretation and highlighting the stopped-flow study of the half-reactions.

The pH-dependency of the TST half-reaction rates indicates that persulfidated TST must be protonated to react with the nucleophilic acceptor, with a p*K*_a_ of 9.38 ± 0.04. The thiol form of TST has a p*K*_a_ of 6.5 ± 0.1 and reacts as an anionic thiolate with thiosulfate. The thiol p*K*_a_ in TST is lower than that of a thiol in a typical peptide (∼ 9.1) (56). Although the assignment of the p*K*_a_ value of 6.5 ± 0.1 to a catalytic residue other than the thiol cannot be excluded, the low value is consistent with modulation by the local environment to favor the thiolate form, promoting the nucleophilic attack on the sulfur donor in the first half-reaction. The low p*K*_a_ is also consistent with a role as leaving group in the second half-reaction, since leaving group potential correlates with acidity. From a structural point of view, the thiol acidity is likely sustained by hydrogen bonds formed between the thiolate and surrounding water, backbone and sidechain groups (57). Regarding persulfidated TST, the available structural information for the bovine enzyme (PDB 1RHD and 1BOH) (Fig. 9) suggests that the persulfide remains in the anionic state due to the establishment of hydrogen bonds (57, 58). The conformational differences between the thiolate and persulfide forms of TST appear to be minimal according to the crystal structures (57). Based on this analysis, it is likely that the p*K*_a_ of 9.38 ± 0.04 corresponds to a different active site residue, which needs to be protonated for the reaction to occur. A potential candidate is Lys_250_ that is located two residues apart from the critical Cys_248_ and has been reported to be important for activity (59). Provision of a positive charge by Lys_250_ would help counteract the negative charges on both the sulfur acceptor substrate and the anionic persulfide.

In summary, our results provide evidence for the existence of the alpha effect in nucleophilic reactions in a series of LMW persulfides and demonstrate that their p*K*_a_ values and rate constants lie within a narrow range, consistent with the substituents being farther away from the outer sulfur than in thiols. Our results also reveal that the low p*K*_a_ values obtained for the LMW persulfides cannot be extrapolated to protein persulfides where the active site environments modulate the acidity and tune the reactivity.

## Experimental procedures

### Reagents, solutions, and buffer systems

Stocks of cystine and homocystine (Sigma) were dissolved in 0.1 M NaOH and used immediately. Solutions of cystamine (Fluka), hydroxyethyl disulfide (Aldrich), and cystine dimethyl ester (Aldrich) were prepared in 0.1 M sodium phosphate with 0.1 mM diethylenetriamine pentaacetic acid (DTPA, Acros). Stocks of H_2_S were prepared from the crystals of Na_2_S·9H_2_O (Carlo Erba or Sigma) stored under argon in a desiccator; they were washed with distilled water and dissolved in ultrapure water the day of the experiment. Concentrated stocks of mBrB (Sigma) were prepared in acetonitrile (AppliChem); dilutions were freshly prepared in buffer and quantified by absorbance at 396 nm (ε_396_ = 5300 M^−1^ cm^−1^) (60). Stocks of 10% 1,2-diheptanoyl-*sn*-glycero-3-phosphocholine (DHPC, Avanti Polar Lipids) were prepared in 10 mM potassium phosphate buffer, pH 6.8. Solutions of sodium sulfite (Sigma), sodium thiosulfate (Amresco), and potassium cyanide (Sigma or Biopack) were freshly prepared in ultrapure water. For TST activity assays, thiosulfate and cyanide were prepared in the assay buffer. Potassium thiocyanate (Fluka) standards were prepared in buffer.

Different three-component buffers with constant ionic strength (*I* = 0.15 M) and variable pH were used, depending on the experiment (61). The acetic/MES/Tris buffer 1× contained 15 mM acetic acid (Dorwil), 15 mM MES (AppliChem or Sigma), 30 mM Tris (AppliChem), 120 mM NaCl (Fluka or Sigma), 0.1 mM DTPA and varying amounts of HCl or NaOH to adjust the pH in the 3.65 to 8.75 range. The MES/Tris/ethanolamine buffer 1× consisted of 20 mM MES, 10.4 mM Tris, 10.4 mM ethanolamine (Sigma), 43 mM sodium sulfate (Sigma), 0.1 mM DTPA, and varying amounts of H_2_SO_4_ or NaOH to adjust the pH in a range of 5.65 to 9.93. The ACES/Tris/ethanolamine buffer 1× contained 30 mM ACES (AppliChem), 15.6 mM Tris, 15.6 mM ethanolamine, 120 mM NaCl, 0.1 mM DTPA, and varying amounts of HCl or NaOH to adjust the pH in the 5.60 to 10.38 range.

### pK_a_ determination of LMW persulfides by the pH-dependence of the reactivities with mBrB

The p*K*_a_ values of CysSSH, HcySSH, CystSSH, β-MESSH, and CysOMeSSH were determined by the pH-dependence of the reaction rates with mBrB, as previously described for GSSH (13).

Persulfide-containing mixtures were prepared by preincubation of the corresponding symmetrical LMW disulfides with substoichiometric amounts of H_2_S for 30 to 60 min at room temperature in sodium phosphate buffer (0.1 M, pH 7.4, 0.1 mM DTPA) (13). Specifically, 3 mM cystine, 5 mM homocystine, 3 mM cystamine, 40 mM hydroxyethyl disulfide, or 2 mM cystine dimethyl ester were mixed with 0.6, 1, 0.6, 8, and 0.4 mM H_2_S, to form mixtures containing CysSSH, HcySSH, CystSSH, β-MESSH, and CysOMeSSH, respectively. The disulfides were chosen based on their commercial availability and on the considerable variations in the p*K*_a_ of the corresponding thiols (16). The concentrations of the disulfides and H_2_S in each case were chosen according to the rate constant of each reaction (5). The formation of persulfides in the mixtures of disulfides and H_2_S was previously characterized by high performance LC-MS of the reaction products of glutathione disulfide and H_2_S (13).

The kinetics of the reactions of LMW persulfides with mBrB were followed in a stopped-flow spectrofluorimeter (Applied Photophysics SX20) with symmetrical mixing under pseudo-first-order conditions with mBrB in excess. One of the stopped-flow syringes contained a 50-fold dilution of the persulfide-containing mixtures (2–6 μM) in ultrapure water, while the other one contained mBrB (100–340 μM) prepared in acetic/MES/Tris buffer 2× with varying pH. The final concentrations were halved by the stopped-flow mixing. The fluorescence (λ_ex_ = 396 nm, emission cut-off 435 nm) of the products was recorded at 25 °C. The final pH values of the reaction mixtures were measured. The data were analyzed with OriginPro 2021.

### SQOR activity assays

Human SQOR was expressed and purified as reported previously (24, 25). SQOR concentration was determined from the FAD absorbance at 450 nm, using ε = 11,500 M^−1^ cm^−1^ (25). Daily stocks of 0.4 μM SQOR were prepared in 50 mM Tris buffer with 100 mM sulfate and 0.03% DHPC, pH 8.0.

The activity of SQOR was measured at different pHs in a temperature-controlled spectrophotometer (Shimadzu UV-2600 or UV-1900i). The steady-state rate of reduction of CoQ_1_ (Sigma-Aldrich or Cayman Chemical) was followed at 278 nm (ε = 12,000 M^−1^ cm^−1^ (25)) in MES/Tris/ethanolamine buffer with pH in the 5.65 to 9.93 range at 25 °C.

In experiments with sulfite as the sulfur acceptor, the reactions contained the corresponding buffer with 69 μM CoQ_1_, 0.03% DHPC, 0.06 mg/ml BSA, 150 μM H_2_S, 1 nM SQOR, and varying concentrations of sulfite (0.01–8 mM), in a total volume of 1.2 ml with minimum headspace. The reactions were initiated by the addition of SQOR. The cuvette was capped during the experiment to avoid H_2_S leakage. At the end of each experiment, the final pH values of the reaction mixtures were measured. The steady-state rates were calculated from the linear fits after SQOR was added; the small slopes before addition of SQOR were subtracted.

Experiments with cyanide as the sulfur acceptor were carried out similarly, but using 90 μM cyanide instead of sulfite and 50 nM SQOR. Reactions were started with cyanide instead of enzyme.

To ensure that CoQ_1_ and H_2_S were saturating at all pH values, the activity of SQOR at the extreme pH values was measured using 0.8 or 4 mM sulfite and 1 or 2 nM SQOR and compared to the activity with higher concentrations of either H_2_S (300 μM) or CoQ_1_ (108 μM). To control for the lack of irreversible inactivation at the extreme pHs, 6 nM SQOR was preincubated for 20 to 40 s in MES/Tris/ethanolamine buffer at pH 5.65, 7.25, or 9.43 with 0.03% DHPC in a total volume of 200 μl. Then, the activity was measured as in a typical assay but using the preincubated enzyme and 0.8 mM sulfite in 82 mM Tris buffer with 82 mM sulfate, pH 7.4 (final concentrations).

Experiments in the presence of chloride were performed as in the regular assay with sulfite as the sulfur acceptor but in the acetic/MES/Tris buffer (pH 7.17), which contained 120 mM NaCl instead of sulfate.

### TST activity assays

Human TST was expressed and purified as described previously (24). The concentration was estimated using an absorption coefficient calculated from the amino acid sequence (ε_280_ = 53,400 M^−1^ cm^−1^) (62).

The activity of TST was measured at different pH values and 25 °C by the steady-state rate of thiocyanate formation. The enzyme (5–100 nM) was reacted with 300 mM thiosulfate and 300 μM cyanide in ACES/Tris/ethanolamine buffer in a pH range of 7.03 to 10.12. After 30 to 90 s, the reactions were stopped by removing 305 μl aliquots and mixing them with 20 μl of 38% formaldehyde (Biopack) and 25 μl of 140 mM FeCl_3_·6H_2_O (Sigma-Aldrich) diluted in 32.5% HNO_3_ (Dorwil). Absorbances at 460 nm (Varian Cary 60, Agilent) were recorded immediately and interpolated into a 0 to 30 μM thiocyanate calibration curve. The thiocyanate standards were prepared daily in ACES/Tris/ethanolamine buffer, pH 7.8, and underwent the same procedures as the samples (1).

To ensure that the thiosulfate concentration was saturating and that the cyanide concentration was below the *K*_m_^cyanide^, TST activity was measured at the most acidic and alkaline pHs tested, using 400 mM thiosulfate and 300 μM cyanide or 300 mM thiosulfate and 150 μM cyanide, at pH 7.1 and 9.7. To control for the lack of irreversible inactivation, 5 nM TST was incubated with 300 mM thiosulfate and 300 μM cyanide at pH 7.1, in a total volume of 1.39 ml. After 220 s, 5 μl of 5 M NaOH were added, changing the pH to 8.5. The concentrations of thiocyanate produced at 252 and 310 s were measured as described.

### Stopped-flow kinetics of TST reactions

The pH-dependency of the reaction rates of TST persulfide with sulfur acceptors and of TST thiol with thiosulfate were studied by following changes in the intrinsic fluorescence (λ_ex_ = 295 nm, US 360 nm bandpass filter) in the stopped-flow spectrofluorimeter.

To prepare persulfidated TST, stocks (∼ 20 μM) were incubated with ∼ 150 μM thiosulfate for 15 min at room temperature. The remaining thiosulfate and the formed sulfite were removed with a PD MidiTrap G-25 column (Cytiva) equilibrated with 10 mM Tris buffer, pH 8.5. The persulfidated TST was diluted in ultrapure water in one of the stopped-flow syringes (1.6–2.0 μM, ∼ 2 mM Tris) and mixed with sulfite (30 and 150 μM) or cyanide (50 and 200 μM) prepared in the other syringe in ACES/Tris/ethanolamine buffer 2× in a pH range of 5.60 to 10.38, at 25 °C. The final concentrations were halved as a consequence of the stopped-flow mixing. The increase in intrinsic fluorescence due to thiol formation was recorded during 0.025 to 10 s depending on the time course. The final pH of the reaction mixtures was measured.

To prepare TST thiol, ∼ 100 μM thiosulfate was added to TST stocks (∼ 20 μM) at room temperature. After 15 min, ∼ 200 μM cyanide was added and left to react for 10 min. The remaining LMW compounds were removed with a PD MidiTrap G-25 column equilibrated with 10 mM Tris buffer, pH 8.5. The TST thiol was diluted in ultrapure water (1.8–2.0 μM) in one of the stopped-flow syringes and reacted with thiosulfate (400 μM) prepared in the other syringe in either acetic/MES/Tris buffer 2× (pH 3.68–8.75) or in ACES/Tris/ethanolamine buffer 2× (pH 6.68–10.34), at 25 °C. The final concentrations were halved as a result of the symmetrical stopped-flow mixing. The decrease in the intrinsic fluorescence caused by persulfide formation was recorded during 0.8 to 200 s depending on the time course, and the final pH was measured.

## Data availability

All data are contained within the manuscript and in the supporting information.

## Supporting information

This article contains supporting information.

## Conflict of interest

The authors declare that they have no conflicts of interest with the contents of this article.
