# Supplementary material for: Acidity of persulfides and its modulation by the protein environments in sulfide quinone oxidoreductase and thiosulfate sulfurtransferase
Source: J Biol Chem. 2024 Mar 11;300(5):107149. doi: 10.1016/j.jbc.2024.107149 (PMC11039317; doi:10.1016/j.jbc.2024.107149)
Supplement: Supporting Information [file mmc1.pdf]

Supporting Information

Acidity of persulfides and its modulation by the protein environments in sulfide quinone oxidoreductase and thiosulfate sulfurtransferase

Dayana Benchoam[1,2,3], Ernesto Cuevasanta[1,2,4,5], Joseph V. Roman[6], Ruma Banerjee[6], and Beatriz Alvarez[1,2,*]

[1]Laboratorio de Enzimología, Instituto de Química Biológica, Facultad de Ciencias, Universidad de la República, Montevideo, Uruguay; [2]Centro de Investigaciones Biomédicas (CEINBIO), Universidad de la República, Montevideo, Uruguay; [3]Graduate Program in Chemistry, Facultad de Química, Universidad de la República, Montevideo, Uruguay; [4]Unidad de Bioquímica Analítica, Centro de Investigaciones Nucleares, Facultad de Ciencias, Universidad de la República, Montevideo, Uruguay; [5]Laboratorio de Fisicoquímica Biológica, Instituto de Química Biológica, Facultad de Ciencias, Universidad de la República, Montevideo, Uruguay; [6]Department of Biological Chemistry, University of Michigan Medical School, Ann Arbor, 48109 MI, USA.

* To whom correspondence should be addressed: Beatriz Alvarez, Laboratorio de Enzimología, Instituto de Química Biológica, Facultad de Ciencias, Universidad de la República, 11400, Montevideo, Uruguay, 11400, Tel: (+598)25250749, beatriz.alvarez@fcien.edu.uy.

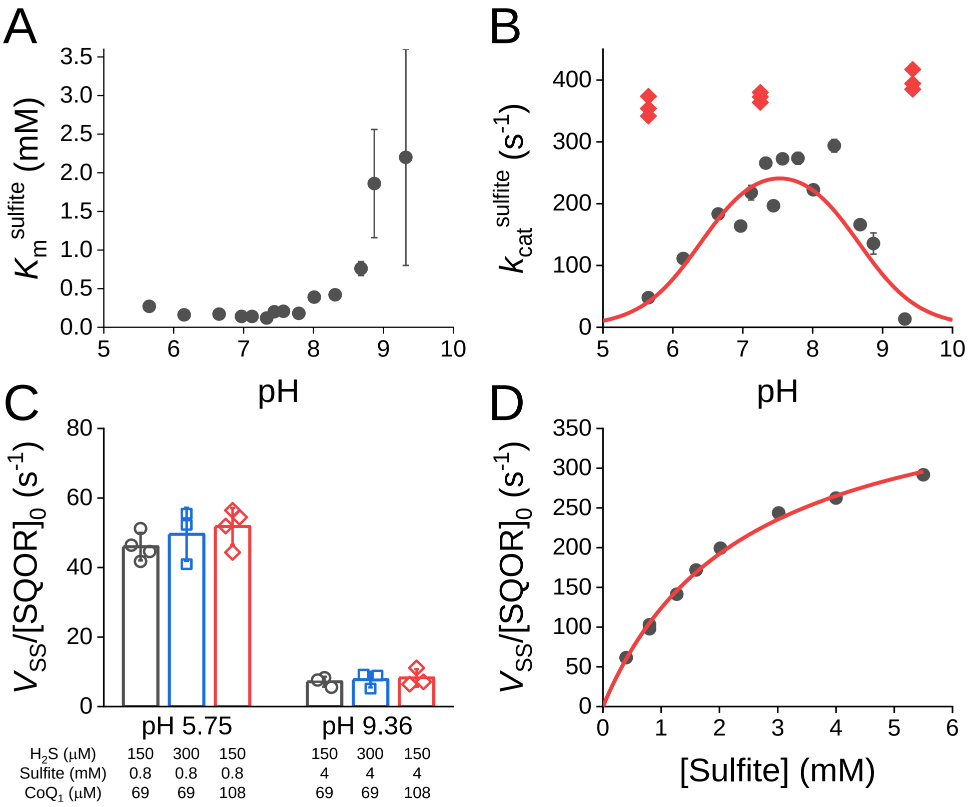

Figure S1. SQOR activity with sulfite as sulfur acceptor. SQOR activity was measured by monitoring the steady-state rate of reduction of CoQ_1 at 25 °C (see Figure 3B). The assays included CoQ_1, DHPC, BSA, H_2S and sulfite, and were started by the addition of SQOR. **(A)** pH-dependence of the $K_m^{sulfite}$ obtained from the hyperbolic fits in Figure 3B ($K_m^{sulfite}$ ± standard errors of the fits). **(B)** $k_{cat}^{sulfite}$ of SQOR *versus* pH. Black circles: pH-dependence of the $k_{cat}^{sulfite}$ in experiments with MES/Tris/ethanolamine buffer, the values were obtained from the hyperbolic fits in Figure 3B ($k_{cat}^{sulfite}$ ± standard errors on the fits). Red diamonds: control for lack of irreversible inactivation at the extreme pHs; SQOR was preincubated for 20-40 s in MES/Tris/ethanolamine buffer at pH 5.65, 7.25 or 9.43. The activities of the preincubated SQOR were measured with saturating sulfite (0.8 mM) in 82 mM Tris buffer, final pH of 7.21, 7.36 and 7.48, respectively. Three independent experiments for each pH were conducted. **(C)** Controls to confirm saturating concentrations of H_2S and CoQ_1 at the extreme pHs. The activities of SQOR with 150 µM H_2S, 69 µM CoQ_1 and 0.8 or 4 mM sulfite at pH 5.75 or 9.36, respectively (black circles) were compared to the activities using 300 µM H_2S and 69 µM CoQ_1 (blue squares) or 150 µM H_2S and 108 µM CoQ_1 (red diamonds). The bars represent the mean ± standard deviation of 3-4 independent experiments per condition. **(D)** SQOR inhibition by chloride. The activity in acetic/MES/Tris buffer containing 120 mM NaCl at pH 7.17 gave an apparent $k_{cat}^{sulfite}$ of (3.1 ± 0.1) × 10^2 s^{-1} and an apparent $K_m^{sulfite}$ of 2.4 ± 0.2 mM (parameters ± standard errors of the fit).

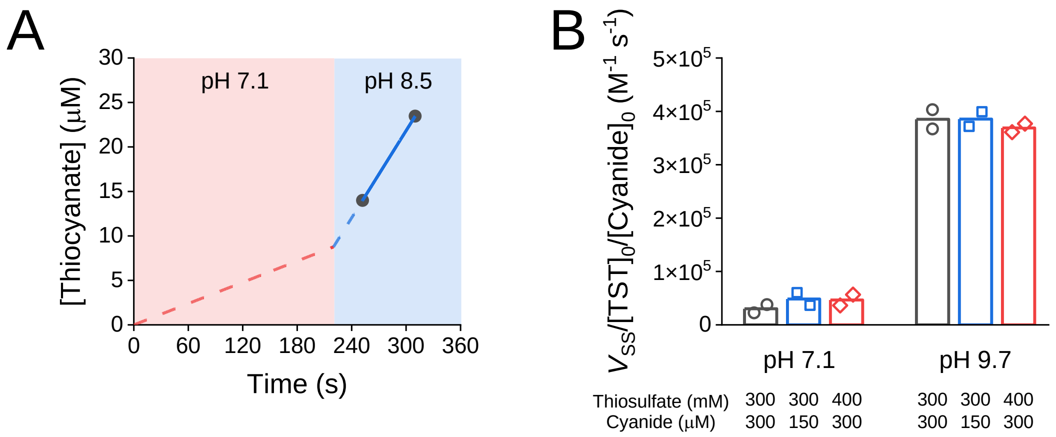

Figure S2. Controls for the pH-dependence of TST activity. The steady-state rate of formation of thiocyanate was measured in ACES/Tris/ethanolamine buffer at 25 °C. **(A)** Control for lack of irreversible inactivation at the most acidic pH tested. TST (5 nM) was incubated with 300 mM thiosulfate and 300 μM cyanide at pH 7.1. After 220 s, NaOH was added to change the pH to 8.5 and the concentration of thiocyanate was measured at 252 and 310 s. The dashed and solid lines represent the estimated rates. **(B)** Controls to confirm saturating concentrations of thiosulfate and concentrations of cyanide below the $K_m^{cyanide}$ at the extreme pH values. The activities of TST (5 or 10 nM) with 300 mM thiosulfate and 300 μM cyanide at pH 7.1 and 9.7 (black circles) were compared to the activities in the presence of 300 mM thiosulfate and 150 μM cyanide (blue squares) or 400 mM thiosulfate and 300 μM cyanide (red diamonds). The bars represent the mean of two independent measurements per condition.
